# Photobiomodulation with Combined Wavelengths Results in Improved Clinical Recovery in a Murine Model of *Bothrops leucurus* Venom Envenomation

**DOI:** 10.3390/toxins17110535

**Published:** 2025-10-30

**Authors:** Gisele Dias da Silva, Fabiana Lessa Silva, Anaiá da Paixão Sevá, Juneo Freitas Silva, Danilo Machado Deorce, Nerildo de Jesus da Costa Junior, Fernanda Amaral Silva, Fernando Alzamora Filho

**Affiliations:** 1 Postgraduate Program in Animal Science, Universidade Estadual de Santa Cruz (UESC), Soane Nazaré de Andrade Campus, Rodovia Jorge Amado, Km 16, Salobrinho, Ilhéus 45662-900, BA, Brazil; giseledias97@hotmail.com; 2 Department of Agricultural and Environmental Sciences, Universidade Estadual de Santa Cruz (UESC), Soane Nazaré de Andrade Campus, Rodovia Jorge Amado, Km 16, Salobrinho, Ilhéus 45662-900, BA, Brazil; apseva@uesc.br (A.d.P.S.); fafilho@uesc.br (F.A.F.); 3 Department of Biological Sciences, Universidade Estadual de Santa Cruz (UESC), Soane Nazaré de Andrade Campus, Rodovia Jorge Amado, Km 16, Salobrinho, Ilhéus 45662-900, BA, Brazil; jfsilva@uesc.br; 4 Medicine Course, Universidade Estadual de Santa Cruz (UESC), Soane Nazaré de Andrade Campus, Rodovia Jorge Amado, Km 16, Salobrinho, Ilhéus 45662-900, BA, Brazil; dmdeorce.med@uesc.br; 5 Veterinary Medicine Course, Universidade Estadual de Santa Cruz (UESC), Soane Nazaré de Andrade Campus, Rodovia Jorge Amado, Km 16, Salobrinho, Ilhéus 45662-900, BA, Brazil; njcosta.mev@uesc.br (N.d.J.d.C.J.); fasilva.mev@uesc.br (F.A.S.)

**Keywords:** *Bothrops* envenomation, snakebite, photobiomodulation, combined-wavelength laser, muscle repair, functional recovery

## Abstract

Snakebite envenomation by *Bothrops* species is a neglected tropical disease and a major cause of local tissue damage and disability in Latin America. Antivenom therapy is effective against systemic effects but fails to prevent local myonecrosis, inflammation, and pain. This study evaluated photobiomodulation therapy (PBMT) using infrared (808 nm) alone or in combination with red (660 nm) laser in a murine model of *Bothrops leucurus* envenomation. A single PBMT session was applied, and animals were evaluated at 24 and 72 h. Combined treatment significantly reduced edema, hyperthermia, plasma CK and LDH, restored nociceptive thresholds, and improved motor recovery compared with infrared alone. Principal component analysis demonstrated clustering of combined-treatment animals with negative controls, supporting a synergistic therapeutic effect. These findings highlight dual-wavelength PBMT as a promising adjunctive approach to antivenom, directly targeting local venom-induced pathology.

## 1. Introduction

Accidental encounters between snakes and humans are a major public health problem, being one of the main causes of morbidity and mortality among all Neglected Tropical Diseases (NTDs) [1,2]. Furthermore, snakebites also impact animal health, affecting companion and production animals worldwide, although underreporting makes it difficult to perceive their importance in this context [3]. Brazil stands out for being one of the countries most affected by snakebite poisoning, with specimens of the *Bothrops* genus responsible for most snakebite accidents, and the *Bothrops leucurus* species predominant in the Northeast Region [4].

The World Health Organization (WHO) has set a target of reducing morbidity and mortality associated with snakebite accidents by 50% worldwide by the year 2030, which highlights the need to develop new studies in this regard [5]. Thus, the search for effective therapeutic modalities in the treatment of victims of snakebite accidents provides an opportunity to explore technological resources capable of enhancing the effects of serum therapy (recommended conventional therapy). Photobiomodulation appears to be a promising tool in the management of local lesions caused by bothropic venom (BV) [6,7], modulating inflammation and promoting faster healing [8,9].

The most evident biological responses observed with the use of laser light for modulation are attenuation of the inflammatory process [10], reduction in biomarkers of damage to muscle cells [11], cytoprotective and myogenic effect [12] and analgesia [13]. The use of laser light to modulate the local effects caused by the venom of snakes of the genus *Bothrops* has been based mainly on the use of red (625 to 745 nm) or infrared (750 nm to 1 mm) wavelengths individually [14,15,16]. Despite this, although studies demonstrate the great potential of this therapeutic modality, the concomitant use of visible and invisible wavelengths has demonstrated more effective effects when compared to their individual use in the treatment of muscle injuries caused by *Bothrops* venom [12,17].

However, previous studies have not evaluated the effects of using red and infrared wavelengths (simultaneously and separately) in reducing local temperature, modulating analgesia, releasing damage-marking enzymes and reducing stress. Therefore, the aim was to evaluate and compare the effects of red and near-infrared wavelengths, used separately and simultaneously, in the modulation of muscle injuries caused by the venom of the snake *Bothrops leucurus* in murine model.

## 2. Results

The results derived from an experimental design that included four groups that received different treatments after intramuscular venom injection: the negative control (NC), the positive control (PC), the infrared laser group (IG, 808 nm), and the combined red and infrared laser group (RIG, 660 and 808 nm). Analyses were conducted 24 and 72 h after a single PBMT session to explore early and late responses. Among these groups, parameters such as edema formation, nociceptive response, local temperature, motor and behavioral performance, and plasma enzyme activity (LDH and CK) were evaluated to characterize the therapeutic effects of each protocol.

### 2.1. Combined Wavelength Treatment Restores Physiological Muscle Temperature Levels

In the thermographic evaluation, there was a significant reduction in temperature in the IG (*p* = 0.02) and RIG (*p* < 0.001) groups compared to the PC group and no significant difference was observed between the treated groups (*p* = 0.38). A significant increase was observed in the PC group compared to the NC group both at 24 h (PC24: 33.4 °C vs. NC24: 27.7 °C; *p* < 0.001) and at 72 h (PC72: 33.3 °C vs. NC72: 27.7 °C; *p* < 0.001). This increase reflects the local inflammatory response induced by the venom.

**Figure 1:**
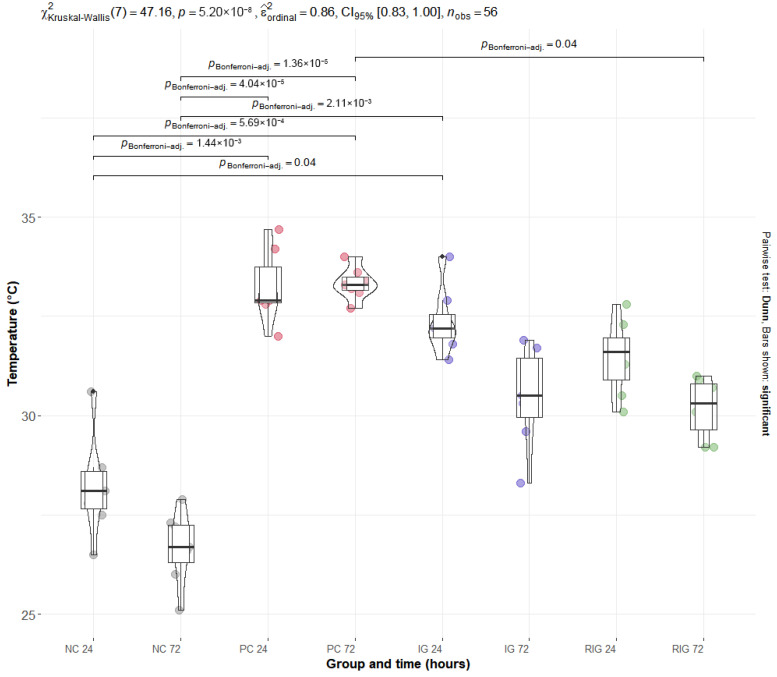
Distribution of thermographic data of the groups Positive control (PC); Negative control (NC); Infrared (IG); and Red plus Infrared (RIG) at different treatment times (*p* values of the Dunn test). Data represent mean values obtained from 10 animals per subgroup (*n* = 10).

Among the treated groups, only RIG showed a significant reduction in muscle temperature at 72 h (30.44 °C) compared to CP (*p* = 0.02). Furthermore, their means were like those of the NC group at both times, indicating a return to baseline levels and suggesting an effective anti-inflammatory effect of the combined treatment. Although IG and RIG showed a downward trend between 24 and 72 h, this intra-group difference was not statistically significant, which indicates that the therapeutic action occurred early and remained stable up to 72 h (Figure 1).

### 2.2. The Association Between Wavelengths Reduces the Edematogenic Activity Induced by the Venom of the Snake B. leucurus

**Figure 2:**
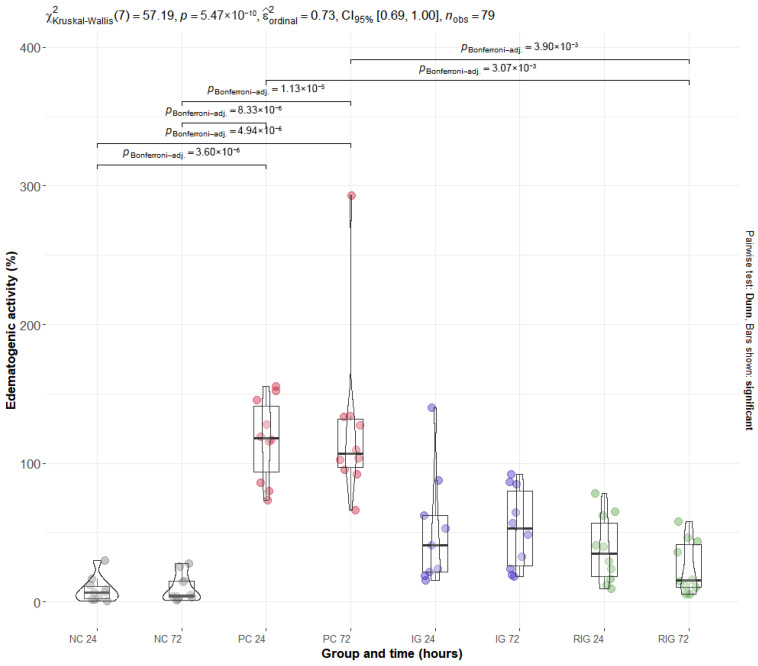
Distribution of edematogenic data in the groups Positive control (PC); Negative control (NC); Infrared (IG); and Red plus Infrared (RIG) at different treatment times (*p* values of the Dunn test). Data represent mean values obtained from 10 animals per subgroup, except for the IG-24 subgroup (*n* = 9) due to one sample being excluded because of technical issues.

BV induced an increase in edematogenic activity in the gastrocnemius muscle, evidenced by the percentage increase in muscle mass. The PC group presented the highest mean values of this variable at 24 h (117.15% ± 29.50) and 72 h (125.77% ± 62.45), with a significant difference in relation to the NC at both times (*p* < 0.001). After 72 h of application, the combination of wavelengths significantly reduced edema compared to PC in both times evaluated (*p* < 0.001), an effect not observed in the IG group. It is noteworthy that the muscle mass values in the RIG group were like those in the NC group at both times, indicating that the combined treatment was able to reverse the edema caused by BV and restore the normal physiological conditions of the gastrocnemius muscle (Figure 2).

### 2.3. The Association Between Wavelengths Reduces the Increased Plasma Levels of CK and LDH Caused by the Venom of the Snake B. leucurus

**Figure 3:**
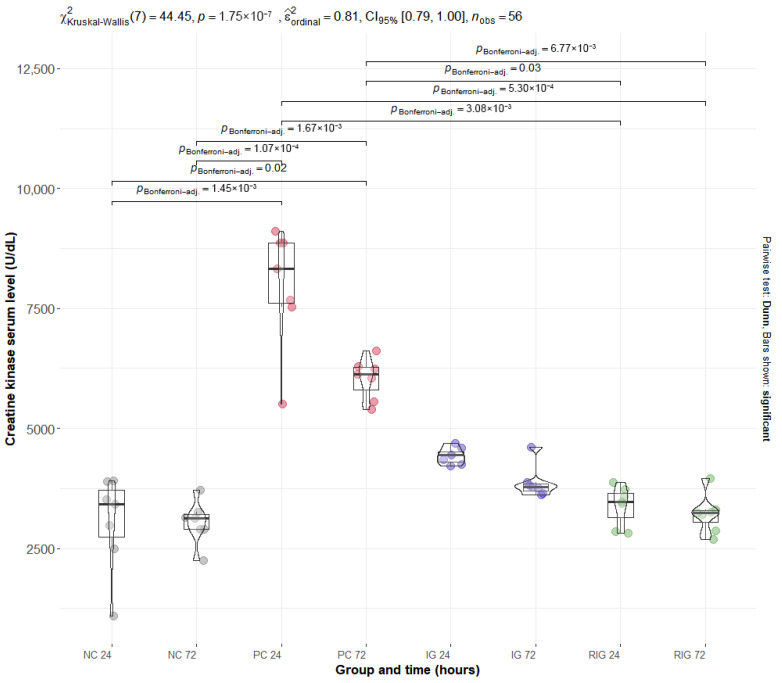
Distribution of serum CK values in the groups Positive control (CP); Negative control (NC); Infrared (IG); Red plus infrared (RIG) at different treatment times (*p* values of the Dunn test). Data represent mean values obtained from 7 animals per subgroup (*n* = 7).

**Figure 4:**
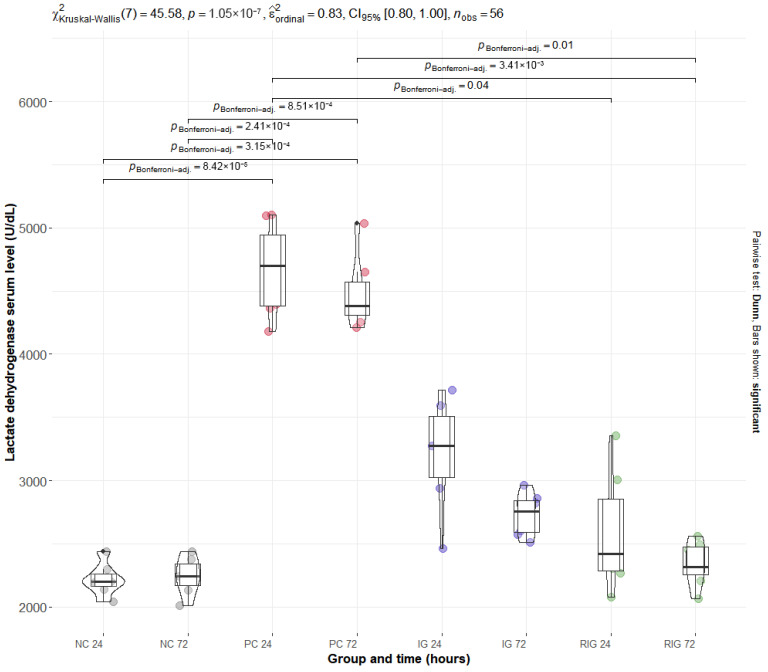
Distribution of serum LDH values in the groups Positive control (CP); Negative control (NC); Infrared (IG); Red plus infrared (RIG) at different treatment times (*p* values of the Dunn test). Data represent mean values obtained from 7 animals per subgroup (*n* = 7).

The mean values of the plasma enzymes CK and LDH were significantly altered by BV, with the PC group presenting the highest levels for both. Multiple comparisons showed a significant difference between the NC and PC groups at both times evaluated (NC24 × PC24 and NC72 × PC72; *p* < 0.001). For the RIG group, a significant reduction in enzyme levels was observed in all comparisons with the PC (NC24 × RIG24, NC24 × RIG72, NC72 × NC72 with *p* > 0.001 and NC72 × RIG24 *p* = 0.03), suggesting a protective effect of the combined treatment on muscle integrity. On the other hand, the IG group did not present statistical differences in relation to the PC, suggesting that the isolated use of a wavelength was insufficient to prevent tissue damage induced by BV (Figure 3—CK; Figure 4—LDH).

### 2.4. Multivariate Analysis Reinforces the Approximation Between the Biological Responses Observed in the NC and RIG Groups

To better understand the effects of treatments on inflammation and evaluate their joint influence on inflammatory parameters (edematous activity, temperature, CK and LDH), PCA was performed. This statistical test allowed us to identify that the variables (parameters) contributed differently to each principal component (PC). Notably, the first component (PC1) explained 80.1% of the total variance of the data, demonstrating that a large part of the information of the variables analyzed can be summarized in this component, and the second component (PC2) explained 9.8%, indicating that, together, they capture almost 90% of the variation in the data.

Furthermore, in PC1, all variables showed positive correlations and similar weights, suggesting that this component reflects a general inflammatory pattern, in which the parameters behave in a coordinated manner. On the other hand, PC2 showed a strong negative association with temperature (−0.82) and a positive association with the other variables, with CK (0.54) having the greatest influence on this component, followed by LDH (0.14) and edema (0.08). This pattern indicates that PC2 is related to an axis of variation associated with muscle temperature, suggesting that after treatment, temperature may fall while the other parameters are still high.

**Figure 5:**
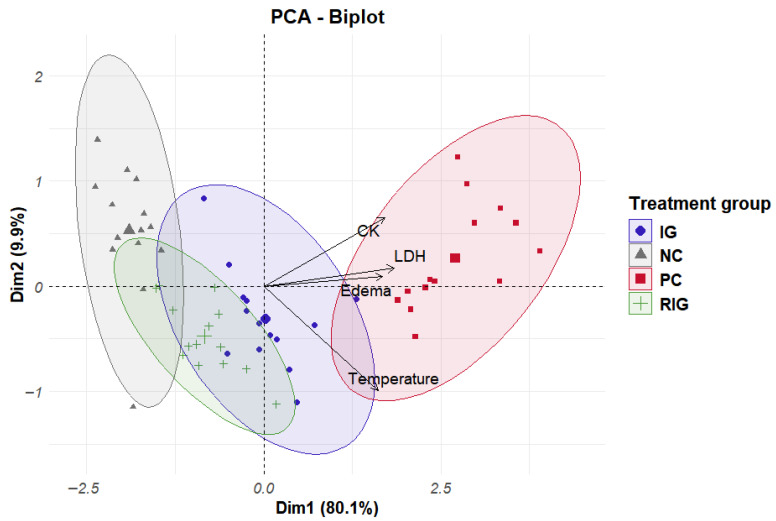
Clustering data from individuals in the groups Positive control (CP); Negative control (NC); Infrared (IG); Red plus infrared (RIG) and relationship between variables and principal components (CP1 = Dim1 and CP2 = Dim2).

The clustering of the data allowed a better understanding of the distribution of the groups in relation to the variables of interest. In Figure 5 in the upper right quadrant, the individuals in the PC group with high values in the variables positively associated with PC1 and PC2 are grouped together. In the lower left quadrant, there are individuals with lower values for these same variables, predominantly from the NC and RIG groups, with interposition between these groups. Thus, it is understood that the PC group presented the highest values for most variables, while the NC and RIG groups recorded the lowest values. Furthermore, the RIG group was closer to negative control when compared to the IG (Figure 5).

It is also worth considering that CK, LDH and edema are variables that respond together in relation to inflammation and treatment. Furthermore, it was possible to observe that individuals in the same group behave in a similar way in terms of inflammatory response for the parameters evaluated, except for the NC group that has an individual with values outside the group standard.

Furthermore, the multivariate analysis of covariance (MANCOVA) indicated a significant overall effect of the treatment factor on the combined physiological variables (Pillai’s Trace = 1.47, F(12,153) = 12.3, *p* < 0.001), demonstrating a strong influence of the treatment on CK, LDH, edematogenic activity, and temperature. Subsequent univariate analyses confirmed significant treatment effects for all variables (*p* < 0.001), with the proportion of variance explained (R^2^) by the treatment ranging from 0.67 to 0.90 (edematogenic activity = 0.67; temperature = 0.78; CK = 0.80; LDH = 0.90). These results indicate that the treatment groups accounted for a substantial portion of the total variation observed in the physiological parameters.

### 2.5. Laser Photobiomodulation Reduces Hyperalgesia Caused by B. leucurus Venom and Associated Wavelengths Bring MNT Closer to Physiological Values

For the evaluation of MNT, the basal threshold of the animals was established prior to the execution of the experimental protocol (13.24 g ± 0.95) and, as expected, no differences were identified between the groups in this period (time 0). After the treatments were carried out, it was observed that the inoculation of the saline solution maintained the threshold close to the basal value in the NC at both evaluation times (12.69 g ± 0.66). In contrast, BV caused a significant reduction in the threshold when untreated (reducing to 8.18 g ± 1.27), whereas, in the treated groups, the values remained closer to those observed in the NC group (RIG—11.35 g ± 1.17, IG—10.60 g ± 2.27).

**Figure 6:**
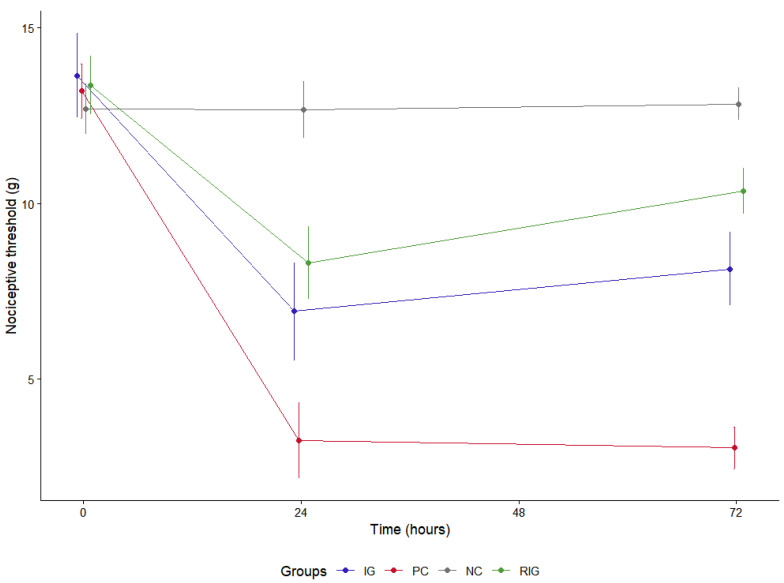
Mechanical nociceptive threshold in the groups Positive control (PC); Negative control (NC); Infrared (IG); Red plus infrared (RIG). There were no differences between the groups at 0 (*p* = 1.000). The NC group differed from all the others at both treatment times (*p* < 0.001). The IG and RIG groups differed from the PC at 24 and 72 h (*p* < 0.001). At 72 h there was a difference between the IG and RIG groups (*p* < 0.001). Data represent mean values obtained from 10 animals per subgroup (*n* = 10).

The IG and RIG groups presented significantly higher thresholds compared to the PC group at both times evaluated (*p* < 0.05). Furthermore, the treated groups differed significantly from each other only at the 72-h time point (*p* < 0.05), with RIG exhibiting the highest values. In the intragroup analysis, the PC, NC and IG groups did not show significant differences between the 24 and 72-h time points, while RIG showed a significant increase in LMN at 72 h after the photobiomodulation session (Figure 6).

### 2.6. Pole Test Reveals Acute Motor Deficit Induced by Bv and Functional Improvement Associated with Laser Photobiomodulation

The pole test was used to assess the motor activity of the animals based on the time taken to turn at the pole (T Turn) and the time taken to lower the apparatus (T Total), so that a longer time means that the reflexes are weaker and the animals may be in a situation of discomfort. For this variable, the data relating to NC were considered as T0, since the saline solution did not cause motor changes in the animals.

**Figure 7:**
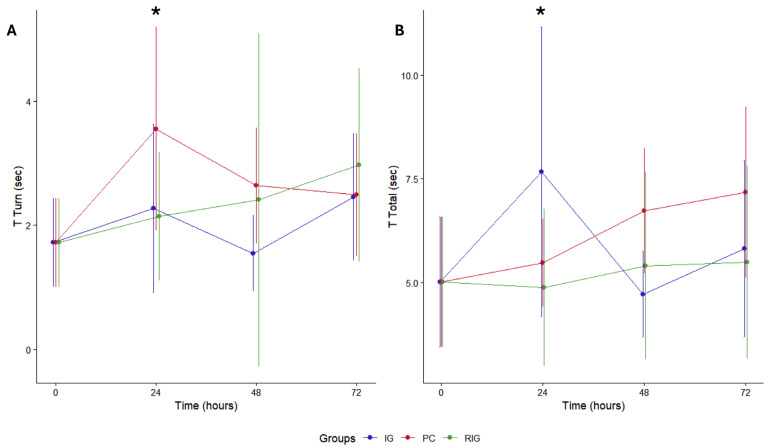
Pole test in the groups Positive control (CP); Negative control (NC); Infrared (IG); Red plus infrared (RIG). Comparison between turn times (**A**) and descent times (**B**). When evaluating the T turn, it was found that the PC group differed from all the others at 24 h after treatment (CP × IG *p* = 0.033; CP × RIG *p* = 0.044). Considering the total T, there was a difference between the PC and the treated groups at this same time (CP × IG *p* = 0.033; CP × RIG *p* = 0.044). (*) indicates *p* > 0.05. Data represent mean values obtained from 10 animals per subgroup (*n* = 10).

The evaluation showed a gradual increase in total T and a peak at 24 h in T Turn for all groups. The IG and RIG groups presented lower T Turn values, with significant differences only between the CP at 24 h (*p* = 0.044) (Figure 7A). The pattern was similar in relation to total T, with differences between the treated groups and the PC at 24 h (*p* = 0.033) (Figure 7B). Thus, it is understood that BV reduced locomotor capacity more significantly in the first 24 h after inoculation and that treatments with photobiomodulation (IG and RIG) helped to attenuate this motor impairment in the same period.

## 3. Discussion

In this study, inoculation with *Bothrops* venom induced characteristic local alterations, including edema, hyperalgesia, myonecrosis, and inflammation, consistent with previous reports in the literature [18]. Photobiomodulation, particularly with combined wavelengths, effectively attenuated muscle damage and local inflammation, as shown by thermography and edema measurements.

It was decided to compare the association between wavelengths with the near infrared wavelength, since most studies indicate that no significant differences are observed between red and infrared in this context and that the latter is used more frequently for the treatment of muscle injuries [6,19]. The results showed that the combined wavelengths produced responses equivalent to those of the negative control group, which reinforce the effectiveness of the photobiomodulation protocol tested and corroborate the initial hypothesis.

Photobiomodulation promoted a reduction in tissue temperature in the treated groups, with the anti-inflammatory effect being more evident in the RIG group. These findings corroborate previous evidence that the therapy does not induce significant thermal increases in tissues [20] and support its effectiveness in attenuating the inflammatory response to BV. Thermographic analysis proved particularly useful in this context, as it is a practical, non-invasive, and effective tool for monitoring musculoskeletal injuries [21,22].

The temperature elevation observed in the PC group is consistent with the inflammatory response triggered by BV, since thermal increase is one of the cardinal signs of inflammation [23]. Indeed, thermography can detect temperature rises ranging from 1 to 5 °C in inflamed tissues [24], reinforcing its potential as a method to monitor the treatment of local lesions caused by BV, also clinically suggested by Carvalho et al. [9].

These findings align with previous studies demonstrating that PBMT reduces local inflammatory responses, which likely contributes to the lower tissue temperatures observed in treated groups. Santos et al. [25] employed a 685 nm wavelength to irradiate cells exposed to BthTX-I, a phospholipase A_2_ myotoxin from *Bothrops jararacussu* venom, resulting in reduced edema and inflammatory markers. Franco et al. [26] applied single wavelengths of 660 nm and 780 nm, separately, to irradiate endothelial cells exposed to *Bothrops jararaca* venom, resulting in decreased IL-1β levels.

Similarly, Oliveira et al. [27] demonstrated, through infrared thermography, that PBMT effectively modulated cutaneous temperature in a neuropathic pain model, indicating a thermoregulatory effect associated with the reduction of inflammatory mediators. This evidence reinforces that the decrease in tissue temperature observed in the present study may reflect the local anti-inflammatory and thermoregulatory actions of photobiomodulation.

The edematogenic response induced by BV was consistent with previous reports and is mainly associated with the action of phospholipases A_2_, metalloproteases, and serine proteases, which promote vascular permeability and fluid extravasation [2,10,28,29]. In the IG group, no significant reduction was observed compared with the PC group, in line with studies indicating that the anti-edematogenic effect of infrared light is more evident within the first hours post-inoculation but tends to diminish at 24 h after a single application [8,30,31,32].

Conversely, the RIG protocol significantly attenuated edema formation at both analyzed times. This enhanced effect is likely related to the combined photobiological actions of vasodilation, increased lymphatic drainage, and cytokine modulation, including reduced expression of IL-1β and IL-18 and increased IL-10 levels [4,12,20]. In addition, PBMT may also stabilize vascular integrity via upregulation of vascular endothelial growth factor receptor 1 (VEGFR-1) [31] and modulation of endothelial nitric oxide synthase [19], contributing to reduced fluid extravasation.

Moreover, attenuation of oxidative stress and inhibition of prostaglandin and COX-2-mediated pathways likely support the sustained anti-edematogenic effect observed with combined wavelengths [26,27]. These molecular and cellular actions synergistically mitigate both the initiation and maintenance of BV-induced edema, highlighting the superior efficacy of combined red and infrared photobiomodulation in controlling local inflammatory responses.

Serum levels of CK and LDH in the RIG group indicate a cytoprotective effect of photobiomodulation, mitigating muscle damage induced by BV. These results are consistent with Dourado et al. [11], who reported significant reductions in these enzymes between 3 and 24 h following red laser application. In contrast, Lauria et al. [10] observed delayed increases in CK and LDH even when using single red or infrared wavelengths, supporting the hypothesis that the combination of wavelengths, as applied here, enhances protective effects on muscle tissue. In the IG group, a single photobiomodulation session showed a tendency to reduce enzyme levels, but the effect was not statistically significant compared with the PC group, suggesting that treatment efficacy may depend on multiple sessions or shorter intervals between applications, given the dosimetric parameters used.

In the untreated PC group, BV inoculation induced pronounced elevations in serum CK and LDH. CK is a compact enzyme (82 kDa) present in the cytosol and mitochondria of high-energy-demand tissues such as skeletal muscle, and its low molecular weight allows rapid release into the bloodstream following cellular injury, making it a sensitive marker of early muscle damage [33,34]. LDH, a cytoplasmic enzyme widely distributed across tissues including muscle, has a higher molecular weight and typically rises in response to significant cell death and cytoplasmic content leakage [35,36]. The concurrent elevation of CK and LDH in the PC group suggests that BV-induced muscle damage involves both early membrane disruption and cell death processes.

Building on these findings, in vitro and ex vivo studies provide additional evidence of the cytoprotective effects of photobiomodulation. Silva et al. [37] demonstrated that irradiation of C2C12 myoblasts with a 660 nm laser following exposure to *Bothrops* venom reduced cytotoxicity and promoted cellular differentiation, with protective effects observed as early as 3 h and sustained up to 24 h post-exposure. Similarly, Reis et al. [38] applied LED PBMT at 630 nm and 850 nm to murine macrophages exposed to Bothropstoxin-I and II, showing decreased release of cytotoxic markers including CK and LDH at multiple time points, notably at 3, 6, and 24 h after treatment. These findings support the notion that both the magnitude and duration of CK and LDH modulation observed in the RIG group are influenced by wavelength selection and treatment timing.

When considering PCA, it is possible to state that the data regarding edematogenic activity and enzymatic markers followed a pattern of photobiological response, with less variation between the NC and RIG groups, which indicates greater effectiveness of the treatment through the association between wavelengths. Temperature, in turn, generated an axis in the opposite direction to the other variables, indicating an inverse correlation between them, which can be justified by the smaller numerical extension of the values obtained through thermographic analysis and explains the importance of PC2 in relation to the total variation of the data [39].

The MANCOVA results demonstrated a strong overall effect of treatment on CK, LDH, edematogenic activity, and temperature. Univariate analyses showed that the proportion of variance explained by treatment ranged from 0.67 to 0.90, indicating that each variable responds differently to the treatments. These findings support evaluating the variables collectively, complementing the PCA results that also highlighted clear separation among treatment groups.

In the present study, the MNT was most reduced in the PC group, indicating heightened pain sensitivity following BV inoculation [40]. This hyperalgesia is associated with the actions of metalloproteases and phospholipases A_2_, which trigger the release of mediators such as histamine, serotonin, peptides, nucleotides, lipids, and bradykinin, activating H1 and B2 receptors in sensory nerves [41,42]. Further contributions to pain arise from chemical mediators released by damaged tissues and inflammatory cells, including mast cells, macrophages, and neutrophils [43].

The IG and RIG groups maintained MNT values close to those of the NC, with RIG showing the highest threshold at 72 h. Photobiomodulation is believed to modulate nociception by restoring ionic balance through changes in cell membrane permeability, enhancing Na^+^/K^+^-ATPase activity, and generating inhibitory postsynaptic potentials that delay neuronal depolarization [44,45]. Additionally, it reduces BV-induced pain through decreased release of pro-inflammatory cytokines (TNF-α, IL-6), lower expression of c-fos proto-oncogenes in dorsal horn nociceptors, and reduced kinin receptor activity [13].

Notably, the effects of photobiomodulation in the RIG group were potentiated 72 h after the session, emphasizing the importance of early intervention. The combined wavelengths also exhibited longer-lasting effects, which may guide the design of therapeutic protocols according to the type and severity of BV-induced changes. Clinically, this is relevant as local pain is a primary symptom of *Bothrops* envenomation [18,46] and intermittent pain represents a common long-term sequela [47]. Thus, combined wavelengths may provide effective short- and long-term pain control.

Lauria et al. [10] evaluated the antinociceptive effects of PBMT in BLV-induced hypernociception in mice, applying 780 nm and 660 nm lasers at multiple time points post-injection. The 780 nm laser showed earlier efficacy, while the 660 nm laser had delayed onset, yet both maintained antinociception throughout the experiment. These findings highlight wavelength-dependent kinetics and support the superior, rapid, and sustained pain relief observed with combined red and infrared light in the RIG group.

The pole test showed that animals in the PC group experienced a progressive increase in total response and turning times, indicating persistent motor impairment and supporting the hypothesis that BV induces neuromuscular deficits [8,10,48]. In contrast, the RIG group maintained more stable values, suggesting that the combined-wavelength photobiomodulation mitigated venom-induced motor deficits and preserved performance. The IG group showed a peak in total time at 24 h, followed by partial recovery, indicating that single-session treatment may promote inconsistent improvements among individuals. At 72 h, the convergence of times across groups suggests a spontaneous recovery component, highlighting the need for future studies, including molecular and electrophysiological analyses, to clarify recovery mechanisms and validate photobiomodulation protocols.

The pole test has been widely validated as an effective method for assessing motor deficits in mice [49]. The test reliably detects increases in turning and total descent times, reflecting neuromuscular dysfunction. In the present study, the progressive changes observed in the PC group and the preserved performance in the RIG group confirm that the pole test was sensitive and effective in capturing *Bothrops* venom-induced motor impairments and the protective effects of combined-wavelength photobiomodulation.

When considering the analyses, a general trend is observed that the association of wavelengths results in more promising photobiological responses. Previous studies have shown that the simultaneous application of red and infrared wavelengths in the treatment of different muscle alterations results in more effective effects when compared to the isolated use of each wavelength [50,51,52]. Furthermore, other investigations reported clinical improvement and favorable histopathological changes, especially in markers of inflammation and tissue repair, in animals subjected to BV inoculation and treated with the combination of these wavelengths, compared to individual use [12,17].

In addition, future studies should consider potential sex-related differences between male and female mice to better evaluate how such biological factors may influence the response to *Bothrops* venom and to combined photobiomodulation. Moreover, exploring the underlying molecular pathways, including inflammatory mediators, oxidative stress, and signaling cascades, would provide a more comprehensive understanding of the biological mechanisms driving these specific responses.

It is believed that the enhancement of therapeutic effects may be related to different levels of light absorption at the lesion site, since superficial and deep tissues interact differently with different wavelengths [53]. The penetration depth of the laser light beam, among other factors, is influenced by the type of chromophore with which the wavelength interacts most strongly [54]. The red wavelength has a greater affinity with hemoglobin and melanin, while near infrared can interact better with water [55]. Thus, the combined application of these wavelengths can amplify the therapeutic effects, reach different tissue layers and enhance the healing mechanisms observed in this study.

## 4. Conclusions

The results of this study demonstrate that photobiomodulation with a combination of wavelengths (660 nm + 808 nm) with an energy density of 10 J/cm^2^ promotes relevant therapeutic effects in the modulation of local damage induced by BV. The treatment reduced edematogenic activity, improved serum enzymatic profile and thermal recovery, in addition to enhancing functional performance in behavioral and motor analyses. These findings support the hypothesis that the synergistic interaction between different wavelengths can enhance the biological response, favoring cellular processes involved in tissue repair and inflammation control. However, additional studies, especially with histological and molecular approaches, are essential to elucidate the underlying pathophysiological mechanisms and establish optimized clinical protocols for the application of this therapy in *Bothrops* envenomation contexts.

## 5. Materials and Methods

### 5.1. Experimental Protocol

Eighty adult male mice weighing between 25 and 30 g, from the Animal Breeding, Maintenance and Experimentation Laboratory (LaBIO) of the State University of Santa Cruz (UESC) were used. The animals were housed in collective cages (seven mice/box), with food and water ad libitum, controlled temperature of approximately 23 °C and a 12 h light/12 h dark lighting regime. All procedures were approved by the Animal Use Ethics Committee (CEUA) of the State University of Santa Cruz (protocol n° 019/22, approved on 2 September 2022).

Four groups (20 animals/group) were established based on the type of treatment: (NC) negative control; (PC) positive control; (IG) infrared (808 nm); and (RIG) red (660 nm) and infrared (808 nm) associated. The groups were subdivided into subgroups (10 animals/subgroup), considering the time of evaluation of the inflammatory parameters, 24 and 72 h, after a single photobiomodulation session. The parameters evaluated at these two times were: (1) edematogenic activity; (2) analgesia; (3) local temperature; (4) motor and behavioral activity; (5) plasma concentrations of lactate dehydrogenase (LDH) and Creatine Kinase (CK) (the number of animals used in each analysis is indicated in the figure legends).

### 5.2. Venom Inoculation

Venom was collected from adult *B. leucurus* specimens maintained at the UESC snake farm, which operates under a permanent license from IBAMA (no. 22752-5). The mass of the lyophilized crude BV was diluted in sterile 0.9% sodium chloride (NaCl) solution at a concentration of 0.6 mg/kg, corresponding to 15 μg for a 25 g animal, in a final volume of 50 μL. The dose used was previously established based on other studies [12,56], being sufficient to induce exclusively local lesions, without triggering systemic manifestations. To preserve the molecular characteristics, the venom was divided into aliquots and stored at −80 °C. After trichotomy and cleaning with 2% chlorhexidine degerming agent (Riohex^®^, Rioquímica, São José do Rio Preto, Brazil), 50 μL of diluted BV and sterile saline solution were applied to the right and left gastrocnemius muscles, respectively, of the PC, IG and RIG groups, while in the NC group saline solution was administered to both limbs.

### 5.3. Laser Photobiomodulation Session

Thirty minutes after inoculation with BV, the diode laser (ECCO vet^®^ dual pen, Campinas, São Paulo, Brazil) was applied to the same site with a power of 100 mW (both for wavelengths emitted individually and in combination), in continuous application mode (CW), 0.1 cm^2^ spot, and wavelengths of 660 nm (red) and 808 nm (infrared), with the possibility of simultaneous emission of both wavelengths (application mode used in the RIG group). The same energy density was established for all treatment groups (Table 1).

The laser device was manually directed at the muscle and the technique was applied punctually, with only one shot being performed per animal in each application in the IG and RIG groups. The animals in the NC and CP groups were subjected to the same process with the laser turned off [55]. All groups received only one photobiomodulation session, with only the interval established between the application and the evaluation of the parameters of interest (24 or 72 h) varying between the subgroups. To perform euthanasia, the anesthetic overdose technique based on Ketamine (180 mg/kg) and Xylazine (30 mg/kg) was used, administered intraperitoneally.

### 5.4. Assessment of Mechanical Nociceptive Threshold (MNT)

The animals were placed in 10 cm × 10 cm boxes (divided into three smaller areas, one for each mouse) equipped with a metal mesh floor, 50 cm above the bench (Supplementary Material Video S1). For acclimatization, the animals were placed in the structure 30 min before starting the test.

The Assessment of mechanical nociceptive threshold (MNT) was assessed using a digital analgesimeter (EFF-301, Insight Equipment, Ribeirão Preto, São Paulo, Brazil) using increasing pressure application. The device used has a pressure transducer connected to a digital force counter expressed in kilograms/force (kgf), having been defined as the minimum force necessary to provoke the paw withdrawal reflex. This force measurement (in grams) was recorded for each animal after three repetitions and the final value was obtained by averaging the three measurements, with voluntary movements associated with locomotion not being considered as a withdrawal response [57].

The pressure transducer contacted the medial plantar area of the right hind limb using an adapted 0.7 mm polypropylene tip. The stimuli were applied at 5-s intervals. The force value recorded was considered as a threshold for mechanical sensitivity. There was a three-day period of adaptation and training of the animals, which resulted in the establishment of the basal nociceptive threshold for each animal, which was used as a control after the experimental protocol.

### 5.5. Pole Test

To verify motor activity, evaluated the time it took for the animal to descend to the ground after being placed on top of a bar (simulating a post). The mice were placed on top of a 50 cm high vertical bar with a diameter of 1 cm. The post was mounted on a rectangular base stand and placed in the home cage so that mice could climb down to the cage floor (Supplementary Material Video S2). The movement of each animal was recorded for accurate counting, so that the recording began when its turning movement began. Times were recorded using analog stopwatches.

The time from the beginning of the turn to the complete downward turn (Tturn) and to the time until the animal reached the ground (Ttotal) were recorded. The test was repeated three times per animal, with a 30-s interval between attempts to avoid exhaustion, and the average of the repetitions was the final value considered for analysis. However, there were some considerations in cases that differed from this usual pattern: (1) When the animal paused during the descent, the test was repeated; (2) When the animal failed to turn but descended with a lateral body position, Ttotal was assigned to Tturn; and (3) When the animal fell from the pole immediately, the maximum times were assigned to Tturn (10 s) and Ttotal (15 s). Assigning a maximum time reflects that the animal had a severe motor deficit, being unable to complete the task, allowing comparison with animals that performed the test correctly [58].

It is worth considering that the mice were previously conditioned to turn and climb down the pole (snout forward), having been placed on the pole test structure for seven consecutive days in the same way as performed in the experiment. To prevent slipping, the surface of the pole was rough, so it was covered with crepe paper tape.

### 5.6. Thermographic Analysis

To analyze the body surface temperature in the anatomical region where the venom was inoculated, a FLIR Thermal^®^ thermographic camera (E8 Pro—20–550 °C, FLIR Systems^®^, Wilsonville, OR, USA) with an emissivity coefficient of 0.98 was used. Immediately after euthanasia, the animals were placed in ventral decubitus at one meter from the apparatus to be photographed. The FLIR Thermal Studio^®^ software (version 1.9.40) was used to record the average temperature in degrees Celsius (°C) in the right gastrocnemius muscle [59]. For this, a selection area of the same radius (0.1 mm) was established around the lesion of all animals (Supplementary Figure S1).

### 5.7. Edematogenic Activity

To evaluate local edema caused by the venom, the masses of the right and left gastrocnemius muscles were measured individually on an analytical balance. The percentage increase in muscle mass subjected to the effects of the venom (right side) in relation to its contralateral side (left side—control) was considered as the effect of the edematogenic activity caused by the venom in the tissue [55] (Supplementary Figure S2).

### 5.8. Plasma Concentrations of LDH and CK

Biochemical analysis was performed to measure serum lactate dehydrogenase (LDH) and creatine kinase (CK) enzyme levels. For this purpose, blood samples (2 mL) were obtained by cardiac puncture immediately after euthanasia. The collected material was stored in tubes without anticoagulant, which were centrifuged for 10 min. The spectrophotometric technique was used in the Bioplus apparatus (Bio-2000^®^, Barueri, São Paulo, Brazil), with commercial kits LDH-NAC Liquiform and CK-NAC Liquiform (Labtest^®^, Santa Lagoa, Brazil), following the manufacturer’s protocol guidelines.

### 5.9. Statistical Analysis

For quantitative variables such as edematogenic activity, temperature, enzyme concentration and mechanical nociceptive threshold between each group, the normality of the data was initially verified using the Anderson-Darling test. Since these data presented a non-normal distribution, the Kruskal–Wallis test was used, followed by the Dunn post-hoc test, with Bonferroni correction for the *p*-value, considered significant when *p* < 0.05 (Supplementary Material—Table S1).

In order to understand whether these parameters vary concomitantly and in association, Principal Component Analysis (PCA) was performed, configuring an approach that reduced the dimensionality of the data set and identified underlying patterns among the variables evaluated. For this purpose, R software was used with the prcomp function and the “stats” package. Statistical analyses and graphs were generated in R, with the ggstatsplot package, using the ggbetweenstats function [60]. Normality was verified with the nortest package, using R, version 4.4.2 (Supplementary Material—R script S1 e R script S2).

A multivariate analysis of covariance (MANCOVA) was conducted using the Jamovi software (version 2.4) [60] to evaluate the overall effect of treatment groups on all dependent variables simultaneously. Pillai’s Trace was used as the multivariate test statistic, as this method is more robust to violations of normality and homogeneity assumptions. When the Pillai’s Trace test was significant, univariate analyses were subsequently performed for each dependent variable to estimate the proportion of variance explained by the treatment (R^2^). The R^2^ values were calculated as the ratio of the sum of squares for the treatment group to the total sum of squares (treatment + residual).

The data from the pole test were subjected to two-way analysis of variance with repeated measures, considering the factors of treatment and time. When a significant interaction between the factors was identified, post-hoc tests with Bonferroni correction were applied. The significance level adopted was *p* < 0.05. The analyses were performed in the Jamovi software, version 3.6.3, and the graphs were generated in the R software, version 4.4.2 (Supplementary Material—R script S3).

## Figures and Tables

**Table 1:** Therapeutic protocol applied in the different treatment groups: NC) negative control; PC) positive control; IG) infrared group; e (RIG) red + infrared group.

Wavelength	Energy/Point	Time (sec)	Energy Density (ED)	Device Power	Spot	Power Density (PD)
NC	NA	10	NA	NA	NA	NA
CP	NA	10	NA	NA	NA	NA
IG	1 J	10	10 J/cm^2^	100 mW	0.1 cm^2^	1 W/cm^2^
RIG	0.5 J + 0.5 J	10	10 J/cm^2^	100 mW	0.1 cm^2^	1 W/cm^2^

NA—not applicable; J–Joules; sec–seconds.

## Data Availability

The original contributions presented in this study are included in the article/Supplementary Material. Further inquiries can be directed to the corresponding author.

## Supplementary Materials

The following supporting information can be downloaded at: https://www.mdpi.com/article/10.3390/toxins17110535/s1. Video S1: Assessment of mechanical nociceptive threshold (MNT) in mice. The device is applied to the hind paw to measure the minimal force that elicits a withdrawal response, indicating pain sensitivity. Video S2: Evaluation of motor performance in mice using the pole test. Total descent time and turning time are measured to assess neuromuscular coordination and motor deficits. Table S1: Table with raw data. R Scrip S1: Statistical analysis—Clinical variables. R Script S2: Statistical analysis—Principal component analysis. R Script S3: Statistical analysis—Pole test. Figure S1: Macroscopic appearance of the left (non-envenomated) and right (envenomated) gastrocnemius muscles of mice 72 h after inoculation, showing variation in muscle coloration induced by venom across the experimental groups: positive control (PC 72 h), infrared (IG 72 h), and red + infrared (RIG 72 h). Figure S2: Images obtained using a thermographic camera, highlighting the circular area around the lesion to record the average temperature.

## Abbreviations

The following abbreviations are used in this manuscript:

BV	*Bothrops leucurus* venom
PBMT	Photobiomodulation therapy
IG	Infrared group (808 nm)
RIG	Red + infrared group (660/808 nm)
NC	Negative control
PC	Positive control
MNT	Mechanical nociceptive threshold
CK	Creatine kinase
LDH	Lactate dehydrogenase
PCA	Principal component analysis
