# Supplementary material for: Photobiomodulation with Combined Wavelengths Results in Improved Clinical Recovery in a Murine Model of Bothrops leucurus Venom Envenomation
Source: Toxins (Basel). 2025 Oct 30;17(11):535. doi: 10.3390/toxins17110535 (PMC12656360; doi:10.3390/toxins17110535)

Supplementary Figure

Figure S1: Macroscopic appearance of the left (non-envenomated) and right (envenomated) gastrocnemius muscles of mice 72 h after inoculation, showing variation in muscle coloration induced by venom across the experimental groups: positive control (PC 72 h), infrared (IG 72 h), and red + infrared (RIG 72 h).

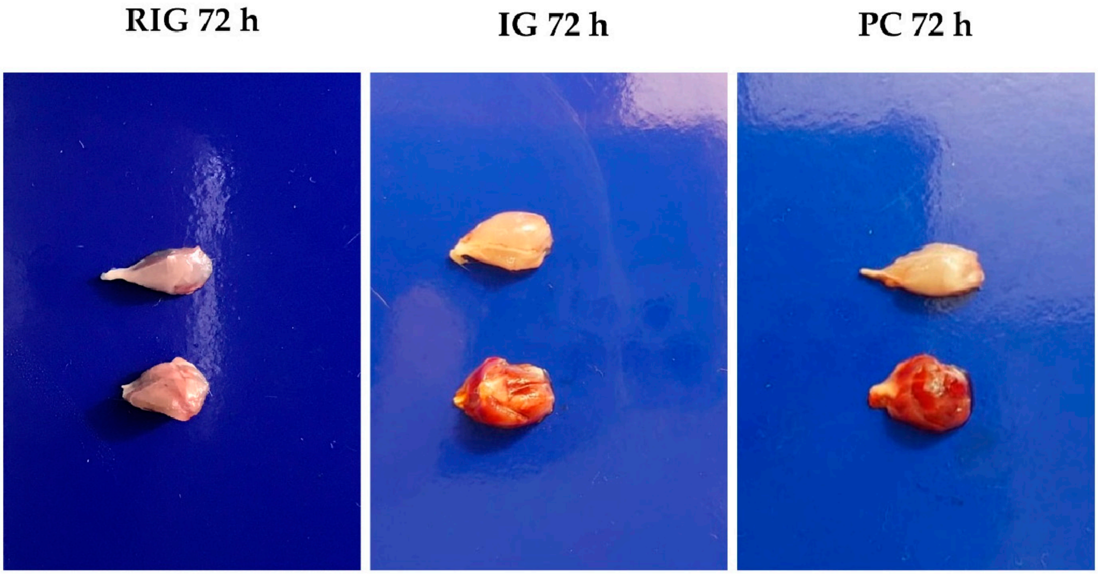

Figure S2: Images obtained using a thermographic camera, highlighting the circular area around the lesion to record the average temperature.

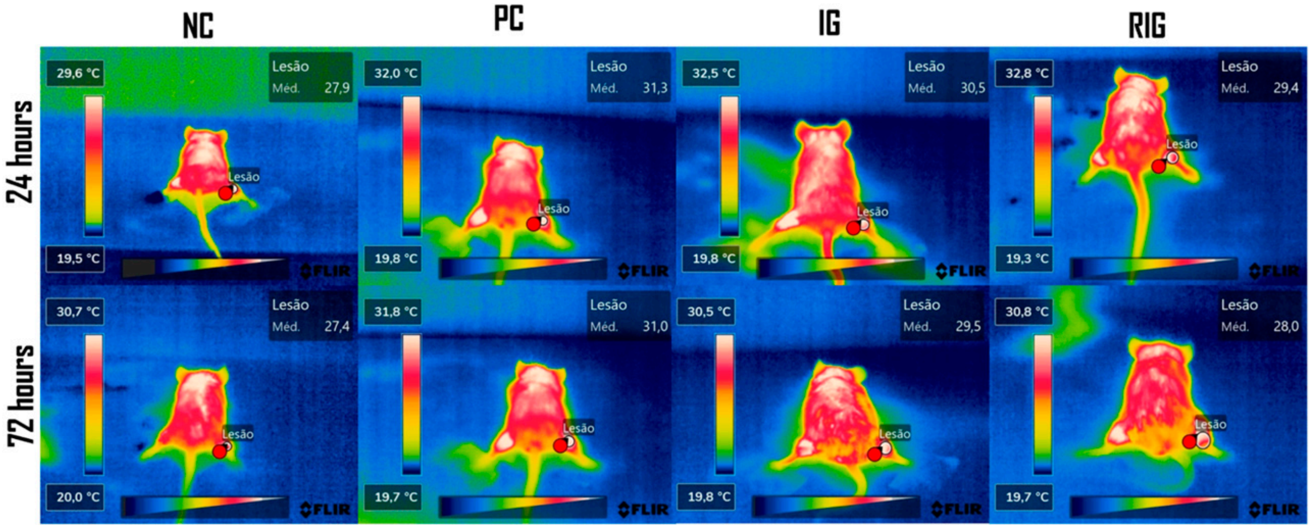

Supplement: Supplementary file 1 [file toxins-17-00535-s001.zip › Supplementary Figure.pdf]
